# Acute Myeloid Leukemia Revealed by a Palatal Necrosis: A Rare Case Report

**DOI:** 10.7759/cureus.32350

**Published:** 2022-12-09

**Authors:** Nouama Bouanani, Houda Bouyaqine, Konimba Coulibaly, Najwa Benslima, Houda Youssefi

**Affiliations:** 1 Department of Hematology, Faculty of Medicine, Mohammed VI University of Health Sciences (UM6SS), Casablanca, MAR; 2 Department of Stomatology and Maxillofacial Surgery, Faculty of Medicine, Mohammed VI University of Health Sciences (UM6SS), Casablanca, MAR; 3 Department of Radiology, Faculty of Medicine, Mohammed VI University of Health Sciences (UM6SS), Casablanca, MAR

**Keywords:** bone marrow aspirate, anthracycline, cytarabine, palatal necrosis, acute myeloid leukemia (aml), oral manifestations

## Abstract

Acute myeloid leukemia (AML) is an aggressive hematological malignancy due to genetic alterations characterized by an overproduction of neoplastic clonal myeloid stem cells in both the bone marrow and peripheral blood. We report a case of a 43-year-old man referred to the department of hematology with a three-week history of palatal pain and weakness. The physical examination revealed an ecchymosis on the left hard palatal mucosa and necrosis. The maxillofacial computerized tomography (CT) scan revealed large osteolysis of the left maxillary bone and a fistulated soft palate. The lesion’s biopsy showed an acute polymorphic inflammation with no sign of malignancy. Laboratory findings revealed anemia, thrombocytopenia, elevated lactic dehydrogenase, and elevated serum ferritin. The diagnosis was subsequently confirmed by a peripheral-blood smear revealing 60% of circulating blasts and bone marrow aspiration with 80% of blast infiltration. The latter was further classified through cytogenetic studies as an AML with deletion of chromosome 7q. This case report aims to highlight the need for clinicians to be aware of palatal necrosis as an initial manifestation of the disease and to emphasize the role of multidisciplinary collaboration between dental surgeons, oral and maxillofacial surgeons, and hematologists for early detection and treatment.

## Introduction

AML is a bone marrow disorder characterized by an overproduction of neoplastic clonal myeloid stem cells and is caused by genetic changes in blood cell precursors. The syndrome usually manifests in adults with a slight male preponderance and a median age of 68 years [1]. The underlying pathogenesis is yet to be determined, but the vast majority are caused by acquired genetic changes, such as isolated gene mutations or chromosomal abnormalities, without clear causative agents [2]. Clinically, patients with AML may present pancytopenia-related signs and symptoms such as infections, hemorrhagic findings, and anemia, as well as tumoral signs. Most notably, the disease's first clinical manifestations may be in the oral cavity. The latter can be found mainly in the oral soft tissues, particularly the gingiva. Gingival bleeding, gingival hyperplasia, ulcers, and petechiae are some of the first oral signs of AML [3]. Moreover, these oral manifestations may have characteristics similar to those of other systemic diseases or specific oral diseases. The primacy to differentiate oral lesions secondary to AML from other disorders is crucial as these clinical signs may be present in other systemic diseases or specific oral diseases, thus hindering an early diagnosis. A thorough systemic examination and timely investigations are therefore all required to elicit its diverse features and firmly rule out other differential diagnoses [4]. To the best of our knowledge, there is scant literature regarding necrosis of the palate as an initial manifestation of the disease. This case remains a very rare initial presentation of the disease [5]. Before being referred to oncohematology physicians for the definitive diagnosis of leukemia, oral clinical signs continue to be the primary complaint of patients visiting dentists. Herein, we report an uncommon case of a patient with acute myeloid leukemia revealed by palatal necrosis.

## Case presentation

**Figure 1:**
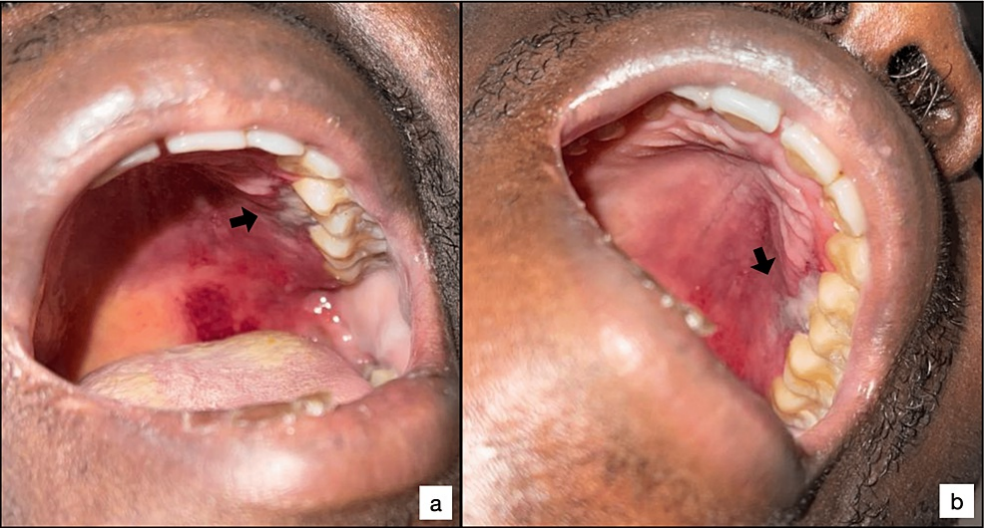
Ecchymosis of the left hard palatal mucosa, with foci of necrosis around the maxillary premolars (arrow)

A 43-year-old man was initially admitted to our hospital with a three-week history of palatal pain. The patient had no other significant personal or family history. Upon general examination, he was conscious with an overall Glasgow score of 15, normotensive, and febrile with a fever of 39°C. Physical examination was notable for painless bilateral axillary lymphadenopathy measuring 1.5 cm with a soft consistency and pallor in the mucous membranes and skin. The intraoral examination revealed an ecchymosis on the left hard palatal mucosa, with foci of necrosis in the maxillary left premolar region, extending from distal lingual 24 to medial lingual 26 and measuring 12mm x 7mm (Figure 1). 

**Figure 2:**
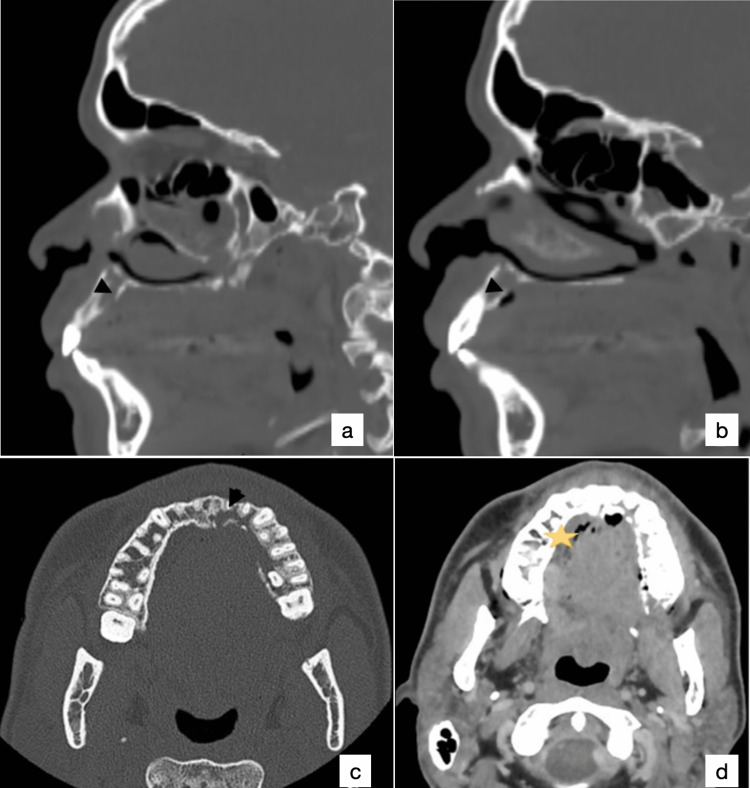
Frontal (a,b) and axial (c: bone window, d: soft tissue window) CT-scan sections showing large osteolysis of the left maxillary bone centered on teeth 21, 22, and 23 (arrowhead) and a short fistula in the soft palate indicated by some air bubbles (asterisk)

The panoramic dental X-ray was normal, but the maxillofacial computerized tomography scan showed large osteolysis of the left maxillary bone centered on teeth 21, 22, and 23 and a short fistula in the soft palate (Figure 2).

After informed consent, a biopsy of the palate was performed. The histological examination revealed readily a non-specific acute polymorphic inflammation of the connective tissue with the presence of granular structures, with no sign of malignancy and no sign of myeloid sarcoma. A microbiological culture was performed, which was non-contributory and ruled out infection, including necrotizing sialometaplasia, tuberculosis, as well as mucormycosis.

Moreover, a urogenital examination was done upon the patient’s request, revealing testicular pain. An ultrasound and a pelvic CT scan were performed, revealing a minimal bilateral hydrocele with scrotal thickening of soft tissues, suggesting scrotal cellulitis.

**Figure 3:**
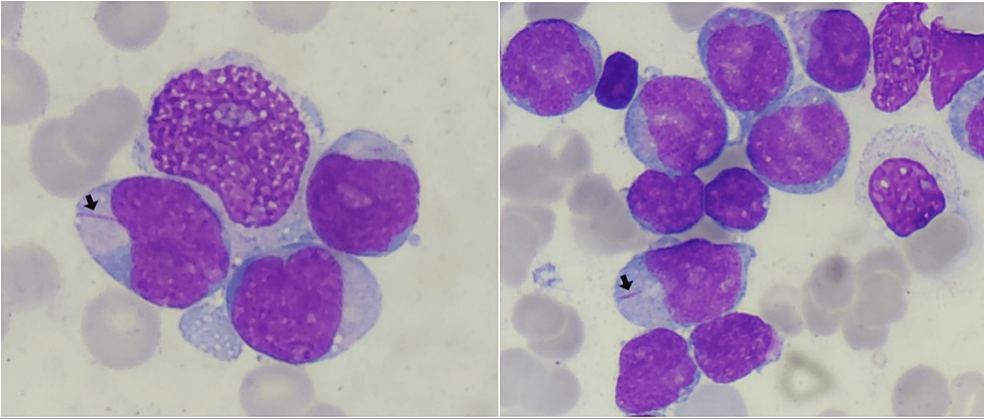
Bone marrow aspirate shows 60% of blast infiltration and needle-sharped cytoplasmic inclusions corresponding to Auer bodies (arrow)

Initial laboratory investigations portrayed normocytic normochromic anemia (hemoglobin of about 10.2 g/dL), leukocytosis (total white cell count of 24.68 x 10³ /mL, neutrophil count of 490/mm³, lymphocyte count of 3460/mm³ and monocyte count of 990/mm³), and thrombocytopenia (platelet count of 80 x 10³ /mL). The peripheral blood smear revealed the presence of 60% circulating blasts. Other blood work tests showed elevated levels of C-reactive protein (CRP) at 15.90 mg/L, lactate dehydrogenase (LDH) at 628 IU/L, ferritin at 1966 ng/dL, and a normal creatinine level at 7.8 mg/L. Further laboratory investigations portrayed normal liver biochemical and endocrine tests. In addition, blood cultures were negative. Extensive research on ongoing infections of the human immunodeficiency virus (HIV), hepatitis panel, Epstein-Barr virus (EBV), syphilis, cytomegalovirus (CMV), and herpes simplex virus (HSV) was deemed negative. Infectious molecular biology and cytobacteriological examination of urine and stools were negative too. No abnormalities were noted in the serum protein electrophoresis and immunofixation. A myelogram was also performed, revealing a very rich amegakaryocytic marrow, 80% invaded by medium to large blasts, with a nucleo-cytoplasmic ratio of 0.8 nuclei. The contours were irregular, with fine chromatin often nucleolated, basophilic cytoplasm showing granulations, and few Auer bodies (Figure 3).

Marrow immunophenotyping confirmed the diagnosis of acute myeloid leukemia. Molecular biology ruled out the presence of the nucleophosmin 1 (NPM1), FMS-like tyrosine kinase 3 internal tandem duplications (FLT3-ITD^high^), and cytosine-cytosine-adenosine-adenosine-thymidine (CCAAT) enhancer binding protein alpha (CEBPA) mutations and cytogenetics studies revealed a deletion of chromosome 7q. Given all of the aforementioned and the updated WHO classification, a definitive diagnosis of AML with deletion of chromosome 7q was made.

The first-line chemotherapy was postponed initially, given the patient's infection and necrosis of the palate. He was therefore administered ceftazidime and amikacin due to his febrile neutropenia. Prophylaxis of fungal infections based on 400 mg per day of fluconazole was administered before undergoing surgery, and the surgical removal was uneventful. Thereafter, the patient received as first-line induction therapy four cycles of the 7+3 chemotherapy protocol using continuous cytarabine at 200 mg/m² on days 1 to 7 and daily anthracycline at 90 mg/m² for the first three days. After chemotherapy, the patient developed once more febrile neutropenia, which was managed with broad-spectrum antibiotic therapy and transfusion support. The patient reached a complete remission (CR) that was confirmed by a myelogram and then underwent three consolidation cycles of cytarabine of 300 mg/m² on days one, three, and five. Two weeks later, the patient left the hospital with upcoming appointments for transplantation. 

## Discussion

AML is a heterogeneous disease due to aberrant differentiation resulting in the uncontrolled proliferation of immature myeloid cells in the bone marrow and peripheral blood. The disease is markedly prevalent in male adults over 60 years of age [6]. Though the underlying pathogenesis is yet to be determined, different risk factors have been associated with this disorder [7]. The updated edition of the WHO classification of hematolymphoid tumors distinguishes two groups of AML: AML with defining genetic abnormalities and AML defined by differentiation and encompasses several changes [8]. Other than the WHO recommendation to classify AML, several other organizations have put forth guidelines to risk-stratify AML. The unifying goal of these guidelines is to delineate cytogenetic abnormalities and mutations, which are important in risk-stratifying patients and therefore determining appropriate treatment. Nearly half of AML patients are cytogenetically normal (CN-AML); however, our patient's cytogenetics studies showed a deletion of chromosome 7q. Moreover, Frederik et al. study suggests an integrative prognostic risk score (IPRS) for CN-AML patients to predict the efficacy of treatment strategies [9].

Clinically, due to bone marrow failure and ineffective erythropoiesis, patients with AML may present a range of symptoms, including bone pain, recurrent infections, and heavy bleeding. As reported in this case, the oral cavity may also be the first site of AML. Acute leukemia, especially AML, is the most prevalent among the various types of leukemia, with oral manifestations as the initial clinical signs. Common findings include gingival bleeding, gingival enlargement, ulcers, petechiae, and candidiasis, but palatal necrosis has been rarely reported [10,11]. These signs could be secondary to neutropenia or direct infiltration by leukemic cells. It is also noteworthy that pre-existing periodontal disease can also exacerbate the leukemic infiltration and worsen oral lesions. Another oral sign that is difficult to evaluate objectively is mucosal pallor. Severely immunocompromised patients may also present bacterial infection, diffused chronic mucocutaneous candidiasis, or recurrent herpetic gingivostomatitis [12].

In our patient’s case, the palate underwent necrosis without any other oral symptoms. The aforementioned signs may be the primary complaint of patients, who therefore see a dentist before being referred to an oncohematology specialist for a definitive diagnosis of AML [13,14]. In previously reported cases, oral soft tissues were the most affected parts in patients with leukemia. While the gingival tissue had the highest manifestation, the mouth floor, parotid, tongue, and nasolabial region had the lowest manifestations of the different types of leukemia [4]. Moreover, it is deemed that bone structures, such as the maxilla and mandible, had the primary characteristics of areas with increased volume, rapid growth, and dental mobility without apparent cause as part of the initial clinical signs of leukemia. These characteristics raised the possibility that the bone lesions were malignant, necessitating further imaging tests to assess them and further referral to a specialized center. Complementary imaging tests like panoramic radiography and computed tomography are necessary to evaluate bone structures more thoroughly, similarly done with our patient [5].

There is scant literature regarding the oral health status and dental conditions of leukemia patients at the time of diagnosis, as most studies focus on the pre-transplantation period in regard to oral lesions secondary to treatment [11]. Altogether, dentists and oral health practitioners’ roles are crucial in the early detection of oral symptoms that are suspicious and, subsequently, in referring patients to hematologists for the diagnosis of leukemia. Their role also extends to the post-treatment and follow-up phase of AML. Additionally, patients should be directed to an oral health program clinic for imaging and any other additional techniques for evaluating oral tissue [15].

It is also important to discard myeloid sarcoma and primary bone marrow necrosis. Of note, acute myeloid leukemia cases make up the majority of myeloid neoplasms that are associated with bone marrow necrosis, in comparison, to chronic myeloid neoplasms. In the case of bone marrow biopsy specimens with necrosis and no indication or history of malignancy, a wide differential diagnosis should be taken into account. Infectious and benign non-infectious causes are both possible. Bone marrow necrosis has a wide range of infectious causes, including bacterial, fungal, mycobacterial, viral, and protozoal infections. Non-infectious etiologies include disorders that lead to thrombosis and vaso-occlusion, like sickle cell disease or antiphospholipid syndrome. Moreover, patients with bone marrow necrosis, in contrast to those with AML, have trouble progressing past the bone marrow aplasia stage [16]. Moreover, myeloid sarcoma is an extramedullary tumor of myeloid cells that frequently arises alongside AML and other hematological cancers. However, its incidence is greater in certain subtypes of leukemia, such as t(8:21) and infant leukemia [17].

Over the past 50 years, the general approach to treating AML has remained largely unchanged. Assessing the risk of treatment-related mortality (TRM) in older patients after intensive therapy is most important, but since TRM rates are decreasing due to better supportive care and better health status in older patients, age is neither the most significant predictor of TRM [18]. In the first-line intensive induction therapy, commonly referred to as the "7+3" protocol using anthracycline and cytarabine, 60% to 80% of younger adults and 40% to 60% of older adults achieve CR, similarly to our patient. Though the 7+3 protocol remains the cornerstone of AML's treatment for eligible patients for intensive chemotherapy, especially those under the age of 60 years, head-to-head trials show that novel therapies outperform the 7+3 regimen.

## Conclusions

Oral manifestations may be the initial sign of leukemia, especially AML. Therefore, oral healthcare professionals should be aware of the oral manifestations of AML and the significance of detecting the signs related to this systemic disease that frequently prompt patients to seek dental care first. To establish an early diagnosis and subsequent treatment, oral practitioners must recognize these signs, investigate them with additional tests, and refer the patient to a specialized center. Furthermore, promoting oral hygiene during the follow-up period is crucial for preventing infections and further complications, including abscesses or sepsis. Oncohematologists, hematologists, dental surgeons and hygienists, and maxillofacial surgeons need to work to encourage early diagnosis and treatment in order to improve patients' long-term oral health.
